# Developing shelf-stable Microbiota Directed Complementary Food (MDCF) prototypes for malnourished children: study protocol for a randomized, single-blinded, clinical study

**DOI:** 10.1186/s12887-022-03436-6

**Published:** 2022-07-01

**Authors:** Ishita Mostafa, Shah Mohammad Fahim, Subhasish Das, Md Amran Gazi, Md. Mehedi Hasan, Kazi Nazmus Saqeeb, Mustafa Mahfuz, Hannah B. Lynn, Michael J. Barratt, Jeffrey I. Gordon, Tahmeed Ahmed

**Affiliations:** 1 Nutrition and Clinical Services Division, icddr,b, Dhaka, 1212 Bangladesh; 2 Faculty of Medicine and Health Technology, University of Tampere, Tampere, Finland; 3 Liggins Institute, University of Auckland, Auckland, New Zealand; 4 The Edison Family Center for Genome Sciences and Systems Biology, Washington University School of Medicine, St. Louis, MO USA; 5 Center for Gut Microbiome and Nutrition Research, Washington University School of Medicine, St. Louis, MO USA; 6 Department of Pathology and Immunology, Washington University School of Medicine, St. Louis, MO USA; 7 Office of the Executive Director, icddr,b, Dhaka, Bangladesh; 8 Department of Global Health, University of Washington, Seattle, Washington, USA; 9 Department of Public Health Nutrition, James P Grant School of Public Health, BRAC University, Dhaka, Bangladesh

**Keywords:** Moderate Acute Malnutrition, Microbiota Directed Complementary Food, Gut Microbiota

## Abstract

**Background:**

Childhood undernutrition is a major public health concern that needs special attention to achieve 2025 global nutrition targets. Moderate acute malnutrition (MAM), manifest as wasting (low weight-for-height), affects 33 million children under 5, yet there are currently no global guidelines for its treatment. We recently performed a randomized-controlled clinical study of a microbiota-directed complementary food formulation (MDCF-2) in 12-18-month-old Bangladeshi children with MAM. The results revealed that MDCF-2, freshly prepared each day, produced a significantly greater improvement in ponderal growth than a standard ready-to-use supplementary food (RUSF), an effect that is associated with repair of the disrupted gut microbial community development that occurs in children with MAM. To test the generalizability of these results in acutely malnourished children at other sites, there is a pressing need for a packaged, shelf-stable, organoleptically-acceptable formulation that is bioequivalent to MDCF-2. This report describes the protocol for a clinical study to evaluate candidate formulations designed to meet these criteria.

**Methods:**

A randomized single-blind study will be conducted in 8-12-month-old Bangladeshi children with MAM to compare the efficacy of alternative shelf-stable MDCF prototypes versus the current MDCF-2 formulation that is produced fresh each day. V4-16S rDNA amplicon and shotgun sequencing datasets will be generated from faecal DNA samples collected from each child enrolled in each group prior to, during, and after treatment to determine the abundances of MDCF-2-responsive bacterial taxa. Efficacy will be assessed by quantifying the change in representation of MDCF-2-responsive gut bacterial taxa after 4-weeks of treatment with freshly prepared MDCF-2 compared to their changes in abundance after treatment with the prototype MDCFs. Equivalence will be defined as the absence of a statistically significant difference, after 4-weeks of treatment, in the representation of faecal bacterial taxa associated with the response to MDCF-2 in participants receiving a test MDCF.

**Discussion:**

This trial aims to establish acceptability and equivalence with respect to microbiota repair, of scalable, shelf-stable formulations of MDCF-2 in 8-12-month-old Bangladeshi children with moderate acute malnutrition.

**Trial registration:**

ClinicalTrials.gov (NCT05094024). The trial has been registered before starting enrolment on 23 October 2021.

## Background

Worldwide, 47 million children under 5-years-of-age are acutely malnourished (wasted), while 149 million are short for their age (stunted) [1, 2]. In Bangladesh, the prevalence of stunting, underweight and wasting among children under 5 were 31%, 22% and 8%, respectively [3]. However, the prevalence of different forms of childhood malnutrition, especially acute malnutrition, is likely to increase as millions of children in low- and middle-income countries around the world are experiencing acute hunger and food insecurity as a result of the ongoing Covid-19 pandemic and related economic turbulence [4]. Moderate acute malnutrition (MAM) in children is defined by anthropometry based on the WHO criteria [(i.e., weight-for-height (WHZ) between 2 and 3 standard deviations below the median of the WHO Child Growth Standards) and/or upper mid-upper-arm- circumference (MUAC) between 115mm and 125mm] [5]. There are currently no global guidelines for the treatment for MAM. Therefore, there is a great need for effective nutritional interventions that could be used to treat undernutrition in infants/children and simultaneously facilitate progress towards achieving the WHO global nutrition targets set for 2025 [6].

Our recent work in undernourished Bangladeshi children has focused on the contribution of the gut microbiota to their nutritional status. Using culture-independent methods to analyze serially collected faecal samples collected from healthy children and from those with MAM, we found that MAM is associated with impaired microbiota development (microbiota ‘immaturity’) [7, 8]. We showed that this immaturity is not repaired by standard nutritional interventions [7, 8]. Furthermore, preclinical studies using gnotobiotic animal models disclosed that colonization with gut communities from undernourished children transmitted impaired weight gain and bone growth phenotypes, plus immune and metabolic abnormalities [9]. Based on these observations, gnotobiotic mice and gnotobiotic piglets were used to screen combinations of culturally acceptable food staples that are commonly consumed as complementary foods in Bangladesh for their ability to increase the fitness of bacterial taxa underrepresented in the immature microbiota of acutely malnourished children. This effort led to the development of several microbiota-directed complementary food (MDCF) prototypes [8, 10]. Three of these formulations were compared to an existing ready-to-use supplementary food (RUSF) in a 1-month randomized controlled ‘pre-POC’ study in 12-18-month-old children with MAM living in an urban slum in the Mirpur area of Dhaka, Bangladesh [8, 10]. One of these formulations (MDCF-2) ‘repaired’ the gut microbial community to state similar to that found in aged-matched healthy Mirpur children, and altered the abundances of plasma proteins in directions that were indicative of improved growth/health status.

We subsequently performed a larger, longer proof-of-concept (POC) study to compare the effects of MDCF-2 and RUSF on the growth of 12-18-month old children with MAM. Despite its lower caloric density (4.30 versus 4.96 kcal/g), children who received MDCF-2 exhibited a significantly faster rate of weight gain during the 3-month period of intervention compared to those consuming RUSF. This improvement was associated with an increase in the abundance of the underrepresented bacterial taxa that MDCF-2 was designed to promote [11].

Both the pre-POC and POC studies described above used a formulation of MDCF-2 that was prepared fresh each day at icddr,b facilities. The objective of the current study is to test industrially manufactured, packaged, shelf-stable formulations of MDCF that would greatly facilitate the expansion of tests of its efficacy to other sites/populations. These studies require development of a uniform formulation that satisfies the following criteria: (i) biological activity equivalent to the current freshly prepared formulation of MDCF-2; (ii) shelf-stable, free of microbial contamination, with satisfactory organoleptic properties; (iii) conforms to existing WHO/UNICEF specifications for a nutritional intervention for treating MAM; (iv) can be produced at scale using ingredients from sustainable sources at an affordable price with the potential for distribution across national boundaries. Here, we present the research protocol for a pre-POC study of candidate shelf-stable MDCF prototypes in Mirpur area of Dhaka city, Bangladesh.

## Methods

### Goal and objectives

The primary goal of the trial is to compare the efficacy of a shelf-stable formulation of MDCF-2 with that of freshly prepared MDCF-2 and assess its organoleptic acceptability. We will also test the extent to which alternative MDCF formulations are able to repair the gut microbiota of children with MAM compared to the repair produced by the current MDCF-2 formulation.

### Study design

**Fig. 1:**
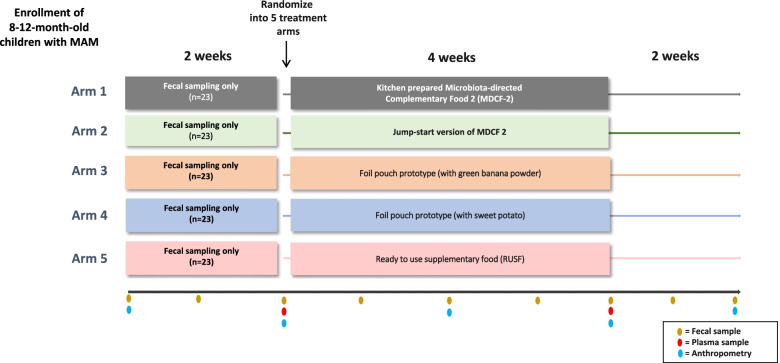
Study design

Figure 1 describes the design of this randomized single-blind pre-POC trial that was approved by the Ethical Research Committee at the icddr,b on 28 June 2021 (PR- 21019).

It will be conducted in three phases. The first 2 weeks after enrolment are a ‘run-in’ phase where children are followed on their normal diets, without nutritional intervention. During this phase, collection of fecal samples will allow the baseline configuration of their microbiota to be characterized prior to treatment. Subsequently, participants will be randomized to receive one of the food prototypes, administered under supervision, twice daily for 4 weeks. Fecal samples collected at the end of the intervention phase will be used to assess the extent of microbiota repair produced by each treatment. After completion of the intervention, each child will be followed for another 2 weeks. Fecal samples collected during this phase will be used to assess the durability of the microbiota repair. Blood samples will be collected at baseline and at the end of the treatment phase to quantify levels of plasma protein biomarkers/effectors of healthy growth [11].

Eligible participants are 8-12-month-old Bangladeshi children with MAM. The study will be conducted among members of the slum population in Baoniabad of Mirpur, one of the 21 administrative units of the nation’s capital, Dhaka. Mirpur was selected because it is inhabited by poor and lower middle-class families, and because residential and sanitary conditions are typical of congested urban settlements. Additionally, the investigators have established several field sites in this area and have ongoing research activities in the community.

### Screening, consent & enrolment of study participants

A trained Field Research Assistant (FRA) will explain the study design and methodology in detail, describe potential risks and benefits, answer any questions from the parent(s), and invite the parent(s) to enroll their child in the study. If the parent(s) are interested, they will proceed to screening and consenting. Eligibility will be determined based on the inclusion and exclusion criteria listed in Table 1.

**Table 1:** Inclusion/exclusion criteria of the study participants

**Inclusion** Parent(s) willing to sign consent form Child aged 8–12 months WLZ score < -2 to -3 without bilateral pedal edema at the time of randomization Parent(s) willing to bring the child to the feeding centre according to the pre-defined schedule **Exclusion** Meeting any of the following criteria will exclude a subject from study participation – Medical conditions: Malnourished children with complications requiring acute phase treatment in a hospital, children with tuberculosis (diagnosis based on WHO 2014 guidelines which have been incorporated in the national TB control guidelines of Bangladesh). The guidelines depend upon the following five diagnostic principles (three out of five should be positive): • Specific symptoms of TB • Specific signs of TB • Chest X-ray • Mantoux test • History of contact Any congenital/acquired disorder affecting growth, i.e., known case of trisomy-21 or cerebral palsy; children on an exclusion diet for the treatment of persistent diarrhea; having known history of soy, peanut or milk protein allergy Severe anemia (< 8 mg/dl) Antibiotic use (within last 15 days before the onset of intervention) Ongoing maternal antibiotic usage for breastfeeding infants Receiving concurrent treatment for another condition	

If a subject is eligible to participate, the consent process will consist of a thorough review of the written consent form in a manner appropriate for the parents’ literacy level. Prior to parental signing of the consent form, the child will undergo thorough clinical examination by study physician. In addition, there may be a recommendation that a child undergo laboratory tests to exclude any organic diseases or any other causes of secondary malnutrition. During this period, parents of the participants will have an opportunity to ask any questions about the study. If the FRA determines that participants have demonstrated adequate comprehension of the study, the consent form will be signed by the FRA and the child’s parent(s). If the parent(s) are not sufficiently literate to read and/or sign the consent form, consenting and a thumbprint signature will be obtained in the presence of a witness who is not associated with the study. The child’s parent(s) will be provided with a copy of the signed consent form.

At the beginning of the study, information will be collected on the demographic characteristics of the participants (family income, standard/type of housing, family structure, parental education, etc.). FRAs will collect anthropometry data by recording the child's weight using a digital scale with 2g precision (Seca, model 728, Germany), length (Seca model 416 infantometer), and mid-upper arm circumference (MUAC) to the nearest millimeter (using a non-stretch tape). Z scores will be calculated using the new child growth standards of the World Health Organization [12]. Collection of anthropometric data will occur at weekly intervals throughout the study.

### Intervention arms

The trial will have 5-arms designed to compare the efficacy of alternative MDCF formulations in repairing the gut microbiota of children with MAM compared to the repair produced by the current kitchen prepared MDCF-2 formulation. All the formulations are fortified with equal amount of vitamins and minerals. Children will be randomly assigned to one of the interventions after enrolment.

- Arm 1 - Reference control: kitchen-prepared MDCF-2
- Arm 2 - Individually packaged, pre-measured sachets of MDCF-2 ingredients, combined and reconstituted in the home setting prior to consumption
- Arm 3 - MDCF-2 shelf-stable foil pouch formulation with green banana powder
- Arm 4 - MDCF shelf-stable foil pouch formulation with sweet potato instead of green banana
- Arm 5 - Ready-to-use supplementary food (RUSF)

Table 2 provides an overview of the interventions.

**Table 2:** Arms and Interventions

	Arms	Intervention
1	Active Comparator: Central kitchen-prepared MDCF-2. Children randomized to this arm will receive 25g of kitchen-prepared version of MDCF-2 twice daily.	Dietary Supplement: Central kitchen-prepared version of MDCF-2. Kitchen-prepared MDCF-2 containing chickpea flour, peanut flour, soybean flour, green banana pulp, sugar, soybean oil and micronutrient mix. This version of MDCF-2 will be freshly prepared in a central kitchen on a daily basis and provided to participants on the same day.
2	Experimental: ‘Jump-start’ version of MDCF-2. Freshly reconstituted MDCF-2 ingredients. Children randomized to this arm will receive individually packaged MDCF-2 ingredients, combined into 21.7 g servings provided twice daily.	Dietary Supplement: Jump-start version of MDCF-2. Freshly reconstituted MDCF-2 ingredients. This version of MDCF-2 is comprised of its individually packaged ingredients. Separate premeasured sachets of chickpea flour, peanut paste, soybean flour, green banana powder, sugar, soybean oil and micronutrient mix will be combined and reconstituted prior to each feeding session.
3	Experimental: MDCF-2 shelf-stable foil pouch prototype with green banana powder. Children randomized to this arm will receive 25g of the shelf-stable foil pouch prototype with green banana powder twice daily.	Dietary Supplement: MDCF-2 shelf-stable foil pouch prototype with green banana powder. This shelf-stable foil pouch prototype of MDCF-2 contains chickpea flour, peanut flour, soybean flour, green banana powder, sugar, soybean oil and micronutrient mix, prepared by an industry partner following the formulation developed at icddr,b.
4	Experimental: MDCF-2 shelf-stable foil pouch prototype with sweet potato. Children randomized to this arm will receive 23.3g of this MDCF prototype with sweet potato twice daily.	Dietary Supplement: MDCF-2 shelf-stable foil pouch prototype with sweet potato. This shelf-stable MDCF prototype contains chickpea flour, peanut flour, soybean flour, sweet potato, sugar, soybean oil and micronutrient mix. This version of MDCF-2 will be prepared by an industry partner following the formulation developed at icddr,b.
5	Experimental: Ready-to-use supplementary food (RUSF). Children randomized to this arm will receive 25g RUSF twice daily.	Dietary Supplement: Ready-to-use supplementary food (RUSF) arm. Locally-produced ready-to-use supplementary food (RUSF) is used as part of a nutritional program to treat moderate acute malnutrition in children over 6 months-of-age. RUSF is eaten directly from the package with no dilution, mixing or cooking. RUSF contains rice, lentil, powdered low-fat milk, soybean oil, sugar, and micronutrient mix.

### Development, delivering & monitoring the interventions

Formulations in arm 1 and arm 2 will be prepared in a kitchen setting. For the rest of the arms, test products will be manufactured in an industrial setting. Each child will receive 250 kcal per day of their assigned formulation. Due to differences in caloric density of the prototypes, the weight per serving of each formulation will be adjusted to ensure equivalent energy provision (Table 2). We have partnered with a large food manufacturer in Bangladesh to develop the shelf-stable, packaged formulations. For this study, we will deliver the industry-made formulations packaged in a trilaminar foil pouch after sterilization. The kitchen-prepared formulations will not be sterilized as these recipes will be delivered immediately after their preparation. A dedicated, BRC- and HACCP-compliant Food Processing Laboratory has been established at the factory site to prepare, package, and sterilize the MDCF and RUSF.

A standardized production procedure is followed throughout the project period to control the quality of the formulations. Raw materials are at first passed through a vigorous quality checking process. The materials are then roasted in a temperature controlled hot air oven followed by a grinding process using a heavy-duty electric grinder to make fine powder/paste as appropriate. To prepare homogenous mixtures, the milled raw materials are shifted to semi-automatic electric mixers to prepare a smooth textured MDCF or RUSF paste. Each step of food preparation, including cleaning, roasting, particle size reduction, and blending, are closely monitored by the study personnel. The paste is then moved to a semi-automatic filler machine in order to fill the trilaminar foil pouch without any direct physical touching by factory personnel. An “Impulse Sealer Machine” is used to achieve airtight sealing of the pouches. Sterilization is performed in a retort machine at 121 °C and 0.208 MPa pressure for a period of 20 minutes. This process ensures a longer contamination-free shelf life while preserving the organoleptic properties of the food. Before distribution to the study site, random samples from each batch will be tested for organoleptic properties and for microbial contamination see Table 3.

**Table 3:** Microbiological and Biochemical test

Parameters for Microbiological tests		
Test name	Method	Acceptable limit CFU/gm
Total plate count/Aerobic bacteria/APC	USFDA/BAM	<10,000
Total coliform/*E. coli*	USFDA/BAM	<10
Yeast and Mold Count	USFDA/BAM	<10
*Salmonella*	USFDA/BAM	Nil
*Shigella*	USFDA/BAM/ ISO	Nil
*Bacillus cereus*	USFDA/BAM/ ISO	<10
*Staphylococci*	USFDA/BAM/ ISO	<10
*Enterobacteriaceae*	USFDA/BAM/ ISO	<10
**Parameters for Biochemical tests**		
Test name	Acceptable limit	
pH	6.5-7.5	
Moisture	<2.5% for dry food product	

Acceptability will be assessed at the feeding center by using the 7-point Hedonic scale. A score of 7, corresponds to ‘like extremely’ while a score of 1 corresponds to ‘dislike extremely’ [13].

### Monitoring food consumption and collection of meta-data

Each child will be offered approximately 25 grams of the diet in each feeding session (Table 2). Feeding will take place under the direct supervision of trained study personnel. The intervention diets are pre-weighed. Mothers will be instructed to collect all unconsumed food for weigh-back so that the amount consumed by each child can be calculated. Only water from the mug is allowed to drunk; the amount of water consumed is also measured. The quantity of MDCF or RUSF consumed will be monitored and calculated in every feeding session. Multiple food frequency data and 24 hours dietary recall data will also be collected.

### Outcome measures

Primary Outcome Measure: Equivalence in the response to MDCF-2 and a test MDCF formulation, based on the change in representation of MDCF-2 responsive gut bacterial taxa after 4-weeks of treatment. V4-16S rDNA amplicons and shotgun sequencing datasets will be generated from faecal samples collected from each child in each group prior to, during, and after treatment to determine the abundances of MDCF-2 responsive bacterial taxa. Equivalence will be defined as the absence of a statistically significant difference after 4-weeks of treatment in the representation of faecal bacterial taxa associated with the response to MDCF-2 in participants receiving a test MDCF, compared to the representation of these bacterial taxa in participants after receiving the reference MDCF-2 formulation for 4-weeks. Other pre-specified outcomes changes in the concentrations of faecal protein biomarkers of gut inflammation [myeloperoxidase (Alpco, Salem, New Hampshire), neopterin (GenWay Biotech, San Diego, California), calprotectin (BÜHLMANN fCAL, Schönenbuch, Switzerland), lipocalin-2 (R&D Systems, USA), dual oxidase 2 (MyBioSource, USA)] and protein losing enteropathy [alpha-1-antitrypsin (Biovendo Chandler, North Carolina)]. These proteins will be measured using commercially available ELISA according to the kit manuals. Changes in oxidation-reduction potential and pH of fecal samples will be quantified using a redox meter (PCE-228-R, PCE Instruments, Southampton, United Kingdom) and pH meter (Hanna Instrument, USA), respectively. In addition, changes in the abundances of 7000 circulating plasma proteins that include biomarkers and mediators of various facets of host physiology will be quantified using the SomaScan-aptamer-based proteomics platform (SomaLogic, Boulder CO, USA).

### Sample size

The sample size was informed by the faecal 16S rRNA gene amplicon datasets generated for the proof-of-concept study of 12 to 18-month-old Bangladeshi children with MAM.^11^ In that study, we identified 209 bacterial Amplicon Sequence Variants (ASVs) that were present above 5 counts in at least 5% of the 939 faecal samples analyzed from 118 participants. We calculated 95% confidence intervals (CI; mean ± 1.96 standard error) for the change (delta) in abundance of each of the 209 ASVs between day 0 (pre-intervention) and 4 weeks of intervention for the 59 children in the MDCF-2 treatment group of the POC study. Using a bootstrapping approach with ASV data from 5000 subsets of 20 children from the MDCF-2 group and 5000 subsets of 20 children from the RUSF group, we identified 121 ASVs whose mean changes in abundance for the MDCF-2 subsets fell within the 95% CI of the mean changes for the 59 MDCF-2 participants, but not in children who had received RUSF, with 80% power (“MDCF-2-responsive ASVs”). Therefore, each arm will include a minimum of 20 children who complete the study. Considering potential drop-outs (15%), 23 children will be recruited per arm.

### Collection, preparation and archiving of biological samples

All biological samples (blood and faeces) will be collected according to the standard operating procedures (SOPs) described in our previous publications [11]. Five millimeters of blood will be collected from each participant before and after the treatment phase (Fig. 1). Whole blood samples will be centrifuged at 3000 x g for 10 minutes to separate the plasma [14]. Aliquots of plasma sample will be stored at -80 °C until analysis.

Faecal samples will be collected weekly during the run-in, treatment and wash-out phases of the study. At the site of collection, samples will be aliquoted into sterile, pre-labelled 2 mL cryovials and immediately placed into pre-charged liquid nitrogen dry cryo-shippers (within 20 minutes of their production by trial participants) for transport back to the laboratory. At the laboratory, vials will be placed on dry ice while they are organized and subsequently transferred to 9x9 freezer boxes. These boxes will be maintained in a -80^o^C freezer prior to analysis. No additives, preservatives or media will be added to the faecal samples.

### Analysis plan

To assess the comparability of microbiota repair elicited by shelf-stable test formulations of MDCF-2 and the reference kitchen prepared formulation, we will sequence PCR amplicons generated from variable region 4 of bacterial 16S rRNA genes present in DNA isolated from faecal samples collected from participants in the different study arms immediately prior to intervention (day 0) and at the end of intervention (day 28). ASV data will be processed using DADA2 [15] and filtered as described [10]. We will calculate 95% confidence intervals of the mean change in abundance of each of the MDCF-2-responsive ASVs identified in this study, between day 0 and day 28, in children who received the reference MDCF-2 formulation, as well as the mean change in abundance for each of these ASVs in participants receiving the test formulations. A test formulation will be considered equivalent to the reference MDCF-2 if >70% of the MDCF-2-responsive ASVs have mean changes in abundance in children consuming a test formulation between day 0 and day 28 that fall within the 95% CI of their mean changes in abundance in the reference MDCF-2 arm. Another embodiment of the analysis of microbial community repair elicited by the reference MDCF-2 and test formulations will involve a comparison of their effects after 28 days of treatment on the abundances of metagenome-assembled genomes (MAGs) identified by shotgun sequencing of the same faecal DNA samples used to produce 16S rDNA amplicons.

Exploratory outcomes will include the effects of the test formulations on features of the plasma and faecal proteomes, including MDCF-2-responsive proteins that are associated with various facets of healthy growth using approaches described in the MDCF-2 POC study [10].

### Current status of enrolment

Enrolment for the study began on 31 October 2021. We are currently recruiting study participants. The enrolment is expected to be completed by 31 December 2022.

### Data confidentiality and archiving

Privacy, anonymity and confidentiality of data will be strictly maintained. All trial related information will be kept confidential and stored securely at the central office in icddr,b. Codes will be used to depersonalise the data. The linking code, electronic data files and paper forms are stored in a separate location under password protections or lock and key. Access to the data will be restricted to the investigators.

## Discussion

The current version of MDCF-2 is freshly prepared in local kitchens on a daily basis and distributed locally on the same day without sterilization. Given the need to identify a bioequivalent formulation that can be produced at scale and distributed to other geographic locales, we have designed this study of children with MAM to test alternative shelf-stable prototypes of MDCF-2 that each provide ~250 kcal/day, plus 70% RDA of key micronutrients. Outcomes will include the organoleptic acceptability of the prototypes and their effects on microbiota repair over 4-weeks of intervention. Our goal is to identify a packaged, shelf-stable formulation for advancing into larger, longer proof-of-concept studies that evaluate efficacy using clinically-relevant growth outcomes. One weakness of this study is that it is not possible to attain full blinding since the test prototypes are not packaged in a similar manner.

In conclusion, this study aims to establish the acceptability of a scalable, shelf-stable formulation that is bioequivalent to MDCF-2 with respect to microbiota repair in 8-12-month-old Bangladeshi children with moderate acute malnutrition. The results of this study will inform the selection of a shelf-stable formulation of its efficacy for treating of moderate acute malnutrition.

## Data Availability

All data related to this study protocol are within the manuscript and its supporting information files. Additional information is available from the corresponding author on reasonable request.

## Abbreviations

MAM: Moderate Acute Malnutrition
MDCF: Microbiota Directed Complementary Food
RUSF: Ready-to-Use-Supplementary Food
MUAC: Mid-Upper Arm Circumference
WHO: World Health Organization
DNA: Deoxyribonucleic Acid
ASV: Amplicon Sequence Variant
